# Functional and Structural Characterization of a Large Animal Model of *RDH5*-Associated Retinopathy

**DOI:** 10.1167/tvst.15.8.2

**Published:** 2026-08-05

**Authors:** Laura M. Ford, Laurence M. Occelli, Mary E. Enfield, Nastassia A. Benjamin, Kelian Sun, Nate Pasmanter, Simon M. Petersen-Jones

**Affiliations:** 1 Genetics and Genome Sciences, Michigan State University, East Lansing, MI, USA; 2 Department of Small Animal Clinical Sciences, Michigan State University, East Lansing, MI, USA

**Keywords:** *RDH5*, inherited retinal diseases, fundus albipunctatus, cat large-animal model, macular degeneration, area centralis degeneration

## Abstract

**Purpose:**

Inherited retinal diseases are a group of hereditary diseases that cause variable levels of blindness and affect a multitude of adults and children. One such disease is fundus albipunctatus (FA). FA is caused by autosomal recessive retinol dehydrogenase 5 (*RDH5*) mutations and results in rod dysfunction leading to night blindness and, in a subset of patients, macular degeneration (MD). We previously reported a spontaneous feline model of FA due to an *RDH5* missense mutation. The affected cats showed rod dysfunction and a proportion developed degeneration of the area centralis (AC), equivalent to MD in humans.

**Methods:**

We used fundus confocal scanning laser ophthalmoscopy and spectral-domain optical coherence tomography imaging, six different electroretinography protocols, immunohistochemistry, and histology/transmission electron microscopy to further characterize this large-animal cat model.

**Results:**

In addition to rod dysfunction, cone recovery from intense stimulation was impaired. For *RDH5^−^^/^^−^* cats that developed AC degeneration, an initial elongation of rod outer segments with disorganization of the distal tips was initially detected in the AC and visual streak, suggestive of impaired shedding/phagocytosis. With progression, photoreceptors degenerated in the AC, matching the MD seen in some human patients.

**Conclusions:**

The *RDH5^−^^/^^−^* cat model recapitulates features of both rod and cone dysfunction seen in human patients with *RDH5* mutations.

**Translational Relevance:**

The *RDH5^−^^/^^−^* cat model offers a unique opportunity to further understand the mechanisms of *RDH5*-associated retinopathies and to investigate potential therapeutic approaches.

## Introduction

Vision begins when a photon of light is focused on the retina and absorbed by a visual pigment, composed of a protein (opsin) and a light sensitive chromophore (11-*cis*-retinal)^1^ located in the photoreceptor outer segments. Phototransduction, or the conversion of light into an electrical signal, has been studied in detail in rods, with photons causing a series of structural changes to 11-*cis*-retinal-opsin in the outer segments. After phototransduction, the visual pigment must be regenerated to allow the photoreceptors to respond to additional photons.^2^ The visual (retinoid) cycle between the retinal pigment epithelium (RPE) and photoreceptors is the classic pathway for regeneration of 11-*cis*-retinal.^3^ An additional cone-specific retina-based visual cycle based in Müller cells is now recognized. Cones’ unique ability to convert 11-*cis*-retinol to 11-*cis*-retinal, likely by the action of retinol dehydrogenase 12 (RDH12), allows for rapid cone recovery after light exposure and for cones to function despite competition for retinoid with rods.^4^^,^^5^

Within the classic visual cycle, following the release of all-*trans*-retinal from the opsin, all-*trans*-retinal is converted to all-*trans*-retinol by RDH8 and transported to the RPE. Within the RPE, lecithin retinol acyltransferase esterifies all-*trans*-retinal to all-*trans*-retinyl esters. The RPE 65 kDa protein (RPE65) then converts all-*trans*-retinyl ester to 11-*ci*s-retinol, which is then oxidized to 11-*cis*-retinal by RDH5. Then, 11-*cis*-retinal is shuttled back to the photoreceptors, where it can bind with photoreceptor opsins to form the light-responsive visual pigment.^6^ The supply of 11-*cis*-retinal to the photoreceptors is necessary for normal vision, and mutations in genes encoding for visual cycle proteins can result in diverse phenotypes including Leber congenital amaurosis, cone dystrophies, rod dystrophies, and cone–rod dystrophies. For example, mutations leading to loss of lecithin retinol acyltransferase or RPE65 function result in a lack of 11-*cis*-retinal production, typically resulting in the severe early-onset condition known as Leber congenital amaurosis. In contrast, mutations in *RDH5* result in a vastly different phenotype with congenital night blindness due to a slow regeneration of rhodopsin.^7^^,^^8^ Unlike the absence of 11-*cis*-retinal production in the RPE due to lecithin retinol acyltransferase or RPE65 function loss, 11-*cis*-retinal is still slowly regenerated with loss of RDH5 function, presumably by the action of other RDHs.^9^ RDH11 and RDH10 have been hypothesized as contributing to 11-*cis*-retinol oxidation.^10^^,^^11^

In addition to night blindness, humans with *RDH5* mutations typically develop white–yellowish retinal lesions radiating out from the macula.^12^^–^^16^ Due to the fundus appearance, the condition is termed fundus albipunctatus (FA). These retinal flecks resemble those seen in another retinal disease, retinitis punctata albescens. However, normal vasculature in FA patients distinguishes them from retinitis punctata albescens patients.^17^ Additionally, these fundus abnormalities resemble vitamin A deficiencies and abetalipoproteinemia. When first described, aberrations in vitamin A or metabolism pathways were theorized as causing FA. In 1999, however, autosomal-recessive *RDH5* mutations were identified as the cause of FA.^8^

Although the slow regeneration of rod function is the main visual impairment, there is growing evidence that FA patients can develop cone dysfunction.^12^ In a subset of FA patients, this leads to macular degeneration (MD) of variable age of onset and severity.^9^^,^^12^^,^^15^ MD can have severe implications on quality of life due to loss of high-acuity vision. This loss results in difficulty in visual tasks such as reading and face recognition and, in severe cases, legal blindness.^18^ The variability in MD penetrance has been speculated to be the result of a modifier gene,^12^ potentially accelerating the degeneration of foveal cones resulting in MD. Although the reduced photopic electroretinogram (ERG) responses are typically correlated with older FA patients with MD, reduced responses have been reported in patients as young as 9 years of age.^19^

*Rdh5* knockout mice have been generated, but fail to recapitulate major features of human *RDH5*-associated retinopathies. A delay in rod dark adaptation is only seen after marked bleaching, suggesting that mice have alternative RDHs active in the biosynthesis of 11-*cis*-retinal.^20^ Additionally, mice do not have a macula-equivalent retinal area, making them poor models for MD. Although the *Rdh5* knockout mouse does not recapitulate features of human disease, we previously described a large-animal model of *RDH5*-associated retinopathy that does recapitulate major features of the human phenotype. These cats have a spontaneously occurring missense variant (c.542G*>*T; p.Gly181Val) in the *RDH5* gene. RDH5 is active as a dimer with Gly181 at the interface between the two RDH5 molecules. The alteration to a Val residue with a large isopropyl side chain is predicted to interfere with dimerization. Because the affected cats do not have detectable RDH5, this dimerization interference likely results in protein degradation. All cats homozygous for the *RDH5* mutation (*RDH5^−^^/^^−^*) have delayed rod recovery compared with wildtype (WT) cats. Degeneration of the area centralis (AC), a region equivalent of the human macula, also develops in many, but not all, of the mutant cats. Some *RDH5^−^^/^^−^* cats show AC changes before 1 year of age, and others fail to develop these changes even when followed to middle age. This difference in AC degeneration phenotypes in our colony of *RDH5^−^^/^^−^* cats occurs despite them being identically housed and fed the same commercial diet. The variability in AC degeneration recapitulates the MD variability seen in humans with *RDH5*-associated retinopathies. Although *RDH5^−^^/^^−^* cats recapitulate both the night blindness and MD phenotype seen in human FA patients, they do not develop the white–yellowish fundus lesions. One explanation for this is species differences. Retinal lesions, like those present in FA patients, are found in humans with other retinal diseases and may be a human-exclusive phenotype (described elsewhere in this article). The phenotypic recapitulation of the *RDH5^−^^/^^−^* cats make them a valuable model for *RDH5*-associated retinopathies.^21^

This study provides further detailed characterization of the *RDH5^−^^/^^−^* cat model, including electrophysiological assessment of retinal function and an ultrastructural and immunohistochemical investigation of the AC degeneration.

## Methods

### Animals

All cats were maintained in the animal housing facility at Michigan State University, kept under 12-hour light:12-hour dark light cycles, and fed a commercial diet. Cats ranging from 2 months to 14 years of age were studied (Supplementary Table S1). All cats used in this study originated from the breeding colony described previously.^21^ Efforts were made, including continued out-breeding, to decrease potential detrimental effects of inbreeding like poor health, infertility, and mortality. All procedures adhered to the ARVO statement for the use of animals in ophthalmic and vision research and were approved by the Michigan State University Institutional Animal Care and Use Committee (PROTO202300089).

### Ophthalmic and Fundus Examinations

Disease phenotype was investigated by routine ophthalmic examination techniques. Wide-angle fundus images were obtained using a Retcam II video fundus camera (Clarity Medical Systems Inc, Pleasanton, CA) and by in vivo confocal scanning laser ophthalmoscopy (Spectralis HRA+OCT, Heidelberg Engineering Inc, Heidelberg, Germany). In vivo cross-sectional retinal images were obtained by spectral domain optical coherence tomography (SD-OCT) imaging (Spectralis HRA+OCT) with all cats under general anesthesia, as previously described.^22^

The follow-up mode was used to acquire images from the same location during longitudinal studies of individuals. Retinal morphology as well as layer thicknesses were assessed. This included specific evaluation of the inner segment/outer segment photoreceptor layers quality (external limiting membrane, ellipsoid zone, and interdigitation zone) and measurement of thicknesses of the total retina; outer nuclear layer (ONL); Receptor+, including the layers between retinal pigmentary epithelium and outer plexiform layer included); and the inner retina (layers between the inner nuclear layer and the internal limiting membrane). These were measured in the four quadrants as well as in the AC. Thicknesses were measured in the center of the AC and 240 and 480 µm superiorly and inferiorly to it.

**Figure 1.**
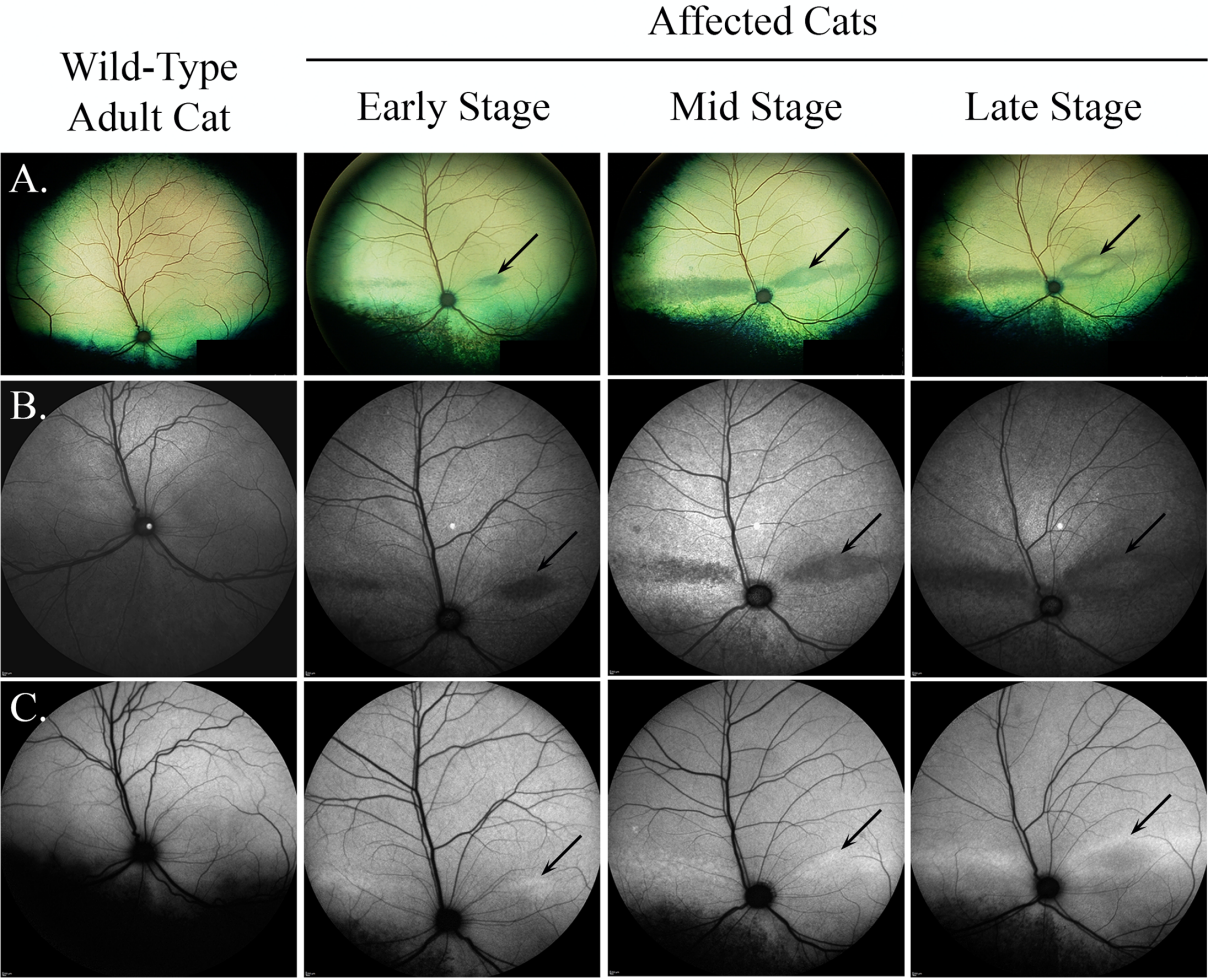
**Representative fundus images of *RDH5^−/−^* cats at different disease stages compared with a normal WT cat.** (**A**) Color fundus photographs, (**B**) infrared (IR), and (**C**) fundus autofluorescence (FAF) confocal scanning laser ophthalmoscopy fundus images. Progressive tapetal reflectivity changes involving the AC (*arrows*) and visual streak develop in a proportion of the *RDH5^−^^/^^−^* cats. In the early stage, on color (**A**) and IR (**B**) imaging the lesions appear as a horizontal hyporeflective band along the visual streak and AC. FAF imaging (488 nm) showed the region to have a mild increase in autofluorescence (**C**). Starting at mid-stage and enlarging in late-stage disease, tapetal hyperreflectivity, indicative of retinal thinning, and reduced autofluorescence have developed in the center of the AC, while the visual streak and the region surrounding the AC region remained hyporeflective with increased autofluorescence. Early stage: *RDH5^−^^/^^−^* cat #0A02104314. Mid stage: *RDH5^−^^/^^−^* cat #0A01681240. Late stage: *RDH5^−^^/^^−^* cat #10-105.

The initial ophthalmoscopic lesion is the development of a hyporeflective streak in the tapetal fundus involving the visual streak and AC (Fig. 1). To investigate diurnal changes on the degree of hyporeflectivity of these lesions, cats with the hyporeflective lesion were examined in the morning within 1 hour of lights on and again in the afternoon after 3:30 pm. Fundus images were captured by a video fundus camera (RetCam II) using identical camera exposure settings for degree of hyporeflectivity comparisons.

### Electroretinography

Cats were prepared for ERG under general anesthesia as previously described^21^^,^^23^ (see Supplementary Table S1 for animal numbers divided by procedure, age, and stage of AC degeneration). Because animals were followed longitudinally with repeated ERGs, the same animal could appear in each age group and each stage of AC degeneration (see Supplementary Table S2 for individual animals used for ERGs). For all ERGs, except the pattern ERG (pERG), ERG-Jet monopolar corneal contact electrodes (Fabrinal Eye Care, La Chaux-De-Fonds, Switzerland) were used.

#### Scotopic (Dark-Adapted) and Photopic (Light-Adapted) ERG

Animals were dark adapted overnight and a dark-adapted luminance series from response threshold to a strong flash was recorded (20 steps ranging from −4.5 log cd⋅s/m^2^ to 1.36 log cd⋅s/m^2^). This was followed by a light-adapted luminance series (12 steps ranging from −2.4 log cd⋅s/m^2^ to 1.36 log cd⋅s/m^2^) after exposure to a 30 cd/m^2^ rod suppressing white light for 10 minutes and followed by a 33-Hz, 3 cd⋅s/m^2^ cone flicker recording, as previously described.^23^

#### Rod Dark-Adaptation Recovery ERG

In addition to the standard dark- and light-adapted protocols, the recovery of rod function after exposure to a rod-suppressing background light was done as previously described.^21^ The cats were dark adapted overnight and a baseline dark-adapted rod ERG recorded. The cats were then exposed to a 30-cd/m^2^ rod-suppressing light for 10 minutes. The background light was switched off and the animals were kept in the dark; recovery of the rod ERG recorded over 100 minutes. This protocol is referred to as the “rod dark-adaptation protocol.”

#### Cone ERG With Repeated Strong Light Exposure (“Bright Flash Protocol”)

To assess changes in cone function with repeated strong light exposure, a protocol was developed that consisted of a rod-suppressing background light with a cone ERG recording (repeated 7.2-cd⋅s/m^2^ flashes) between which the eye was exposed to a set of strong flashes (4 flashes of 1000 cd⋅s/m^2^). This alternating strong light exposure with a cone ERG recording was repeated every 5 seconds (25 times in total) to monitor the effects of cone recovery from the accumulative effects of the strong light stimuli. This protocol is referred to the “bright flash protocol.”

#### Cone Recovery ERG Protocol

After completion of the bright flash protocol, the recovery of cone function was assessed by recording ERG responses to 7.2-cd⋅s/m^2^ flashes every 5 minutes over a period of 125 minutes in the presence of a rod suppressing background light. This protocol is referred to as the “cone recovery protocol.”

#### ON–OFF ERG

In some animals, ON–OFF (long flash, 250 ms, 180 cd/m^2^) recordings were made before the bright flash protocol and then again immediately after the protocol.

#### Pattern ERGs

pERGs were recorded in light-adapted conditions using a Roland Consult RETI-map (Roland Consult, Brandenburg, Germany) and a RM electrode for canines (RetMap Inc, Chicago, IL) with the interior portion of the contact lens removed to allow projection of the pattern on to the retina. The generated checkerboard pattern was centered on the AC*.* A reversal rate of 4.0 reversals per second was used at 0.12, 0.25, 0.50, 0.75, 1.0, and 2.0 cycles per degree (CPD). Each step was averaged across 200 sweeps with 100% contrast. Amplitudes of the P1, N2, N2:P1 ratio, and peak times were measured for comparison (see Supplementary Fig. S1 for a pERG example).

#### Analysis of ERG Waveforms

The a- and b-wave amplitudes and peak times were measured by standard protocols. The Naka-Rushton function was used to fit the first limb of the dark-adapted b-wave luminance-response plot using the equation below:

$$\begin{array}{c} R/V_{m}=L^{n}/\left(L^{n}+K^{n}\right), \end{array}$$

where *V_m_* is the maximum amplitude response, *K* is a semisaturation constant that is considered a measure of retinal sensitivity, and *n* is a dimensionless constant dependent on the slope at position *K*.^24^^–^^26^ Curve fitting was done in RStudio (Version 2023.06.1+524)^27^ using nonlinear least squares (*nls*; R package ‘stats’).

Modeling of the dark-adapted rod a-waves was performed with analyses restricted to flashes ranging from 0.996 to 7.200 cd⋅s/m^2^. Before modeling, photopic matched ERG waveforms were subtracted to assess only the rod driven portion of the a-wave.^28^ The Birch and Hood version of the Lamb and Pugh rod phototransduction model was used to fit the leading edge of the a-wave using the equation below:

$$\begin{array}{c} R=R_{max}\cdot (1-\exp\left[-I\cdot S\cdot {\left(t-t_{d}\right)}^{2}\right]\;for\;t>\;t_{d} \end{array}$$

where the amplitude *R* is a function of the retinal luminance, *I*, and time *t* after the flash. The *t_d_* is a brief time delay, *S* is a sensitivity factor, and *R_max_* is the maximum amplitude.^29^^,^^30^ Parameters were calculated using the *lmfit* curve-fitting program using Spyder 5.5.1.^31^ Goodness-of-fit was calculated, with less than 0.25 considered a good fit. All parameters included in the analysis had a goodness-of-fit value under this 0.25 threshold.

### Immunohistochemistry

After humane euthanasia, eyes were removed and fixed in 4% paraformaldehyde (Electron Microscope Sciences, Hatfield, PA) in phosphate-buffered saline (Sigma-Aldrich, St. Louis, MO) and embedded in optimal cutting media (TissueTek OCT, Electron Microscopy Sciences) for frozen sectioning, as previously described.^32^ Table 1 lists the antibodies used.

**Table 1.** List of Immunohistochemistry Antibodies, Source, Type, and Dilution

Antibody – Source	Type	Primary Dilution	Secondary Antibody – Source	Secondary Dilution
RetP1 (Rhodopsin Ab-1)	Monoclonal mouse	1:2	Alexa Fluor 594 Rabbit anti-mouse IgG	1:500
Thermo Fisher Scientific, Rockford, IL, USA			Life Technologies, Carlsbad, CA, UA	
hCAR (Human cone arrestin)	Polyclonal rabbit	1:10,000	Alexa Fluor 488 Goat anti-rabbit IgG	1:500
Dr. Cheryl Craft; LUMIJ, University of Southern California Los Angeles, CA, USA			Life Technologies, Carlsbad, CA, USA	
RPE65 (Retinal pigmentary epithelium-specific 65kDA protein)Dr. Debra Thompson; Kellogg eye center, University of Michigan, Ann Arbor, MI, USA	Monoclonal mouse	1:500	Alexa Fluor 594 Rabbit anti-mouse IgGLife Technologies, Carlsbad, CA, USA	1:500

### Histology/Transmission Electron Microscopy

Following humane euthanasia, eyes were removed and fixed in 3% glutaraldehyde and 2% paraformaldehyde (Electron Microscopy Sciences) in 0.1 M sodium cacodylate buffer (Electron Microscopy Sciences), as previously described.^23^ After fixation, 3.0- to 3.5-mm diameter biopsy punches were obtained from the AC, visual streak, dorsal, and ventral regions and resin embedded as previously described.^23^ The 500-nm-thick sections were obtained and stained with epoxy tissue stain (Electron Microscopy Sciences) and imaged on a light microscope (Nikon Eclipse 80i, Nikon Instruments Inc, Melville, NY). The 100-nm thin sections were captured on copper grids and stained with 4% uranyl acetate and then Reynolds lead citrate (Electron Microscopy Sciences). They were then imaged on a JEOL 100CX transmission electron microscope with a Gatan ORIUS camera (Gatan Inc, Pleasanton, CA).

### Statistical Analyses

A linear mixed model with random effect of cat and fixed effects of degeneration stage, age group, ERG ‘step’ (flash intensity or time since beginning of ERG), sex, and the interaction between degeneration stage and step was performed in RStudio (*lmer*; ‘lme4’,^33^ and ‘*lmerTest*’^34^ packages) to compare the effect of degeneration stage on different ERG parameters. Least square means of fixed effects were calculated using “lsmeans” in RStudio. Age was binned into three groups: Bin 1, (0,1.5 years), Bin 2, (1.5,3 years), and Bin 3, (≥3 years). Unless otherwise stated, the model above was used for all ERG analyses due to no other significant interactions being detected. The threshold for significance was a *P* value of <0.05. Missing values were removed from the dataset, but datasets that were missing large amounts of data (i.e., some steps of pERG) were interpreted with this in mind.

## Results

### *RDH5^−^^/^^−^* Cats Show Variability in Photoreceptor Degeneration in the AC

The onset of ophthalmoscopic fundus changes in some *RDH5^−^^/^^−^* cats was present as early as 5 months of age, while others did not develop ophthalmoscopic lesions during the study period (up to 7 years of age). For those cats that developed lesions, the initial change was a horizontal gray band of hyporeflectivity in the tapetal fundus encompassing the visual streak and AC (Fig. 1A). This fundus appearance is indicative of an increased absorption of light by the retina overlying the tapetum, infrared confocal scanning laser ophthalmoscopy imaging also highlighted this change (Fig. 1B). The affected region showed increased autofluorescence on fundus autofluorescence imaging (Fig. 1C).

**Figure 2.**
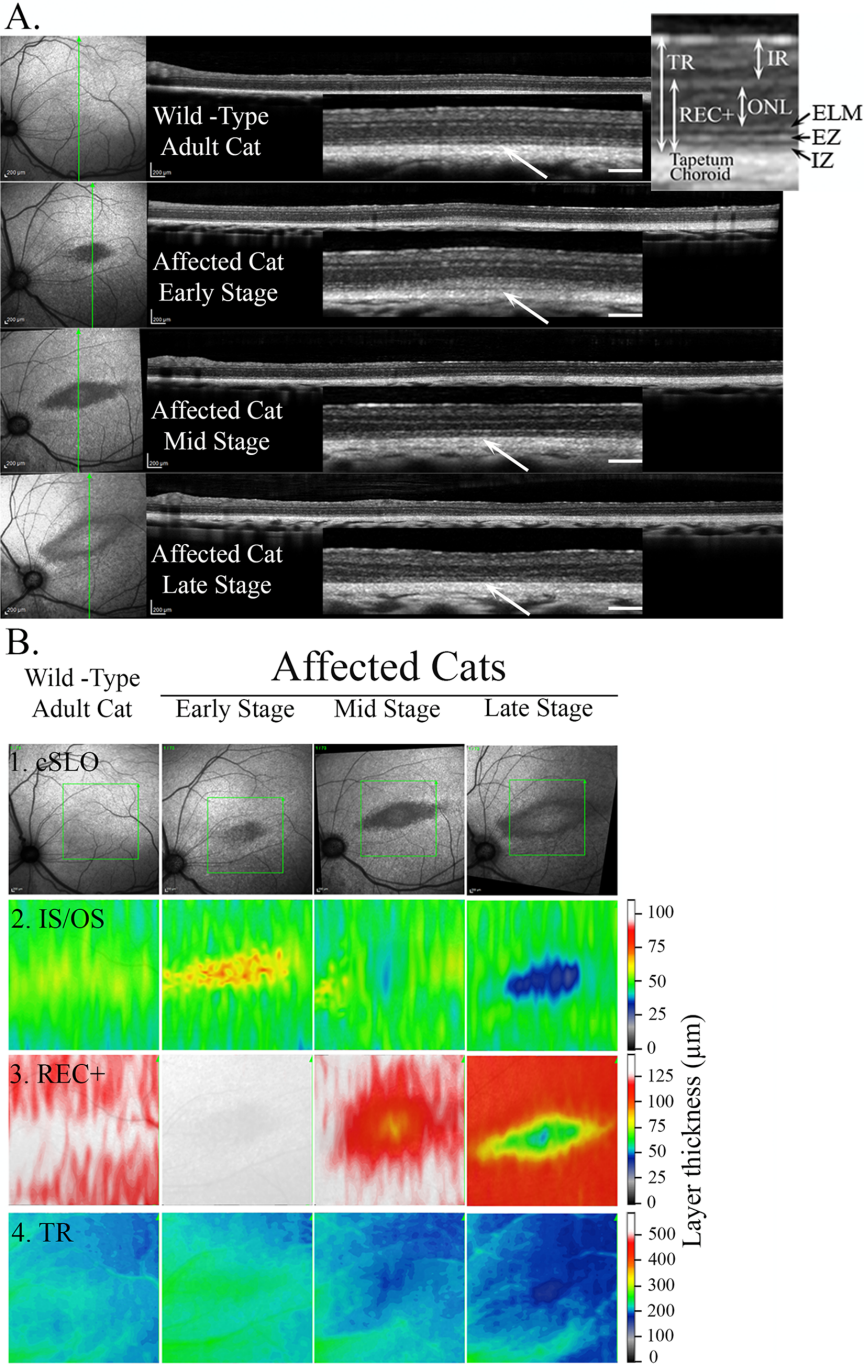
**In vivo cross-sectional imaging of AC lesion.** (**A**) SD-OCT high-resolution vertical cross-section retinal images through the center of the AC (*white arrows*). Location of the retinal cross-sections is indicated by the green line on the confocal scanning laser ophthalmoscopy (cSLO) images on the left panel. *Scale bar*, 200 µm. (**B**) (1) Infrared (IR) cSLO images and (2, 3, and 4) heat maps of the thicknesses of the photoreceptors IS/OS, the REC+ layer and the total retina (TR), respectively, at progressive disease stages. Location used to generate the heat maps is indicated by the green boxes on the top panel cSLO images (1) and encompasses the AC*.* (2) With disease progression the IS/OS progressively thinned in the center of the AC*.* Similar thinning was observed in the REC+ (3) and TR (4) layers. The heat map shows that the thinning starts in the center of the AC then expands to the adjacent retinal area. Early stage: *RDH5^−^^/^^−^* cat #0A02104314. Mid stage: *RDH5^−^^/^^−^* cat #0A01681240. Late stage: *RDH5^−^^/^^−^* cat #10-105. ELM, external limiting membrane; EZ, ellipsoid zone; IR, inner retina, including all layers from the inner nuclear layer to the external limiting membrane; IS/OS photoreceptor inner/outer segments; IZ, interdigitation zone; REC^+^, receptor plus, which includes all layers from the IZ to the outer plexiform layer (OPL), representing the entire photoreceptor cell length; TR, total retina.

The main SD-OCT features of hyporeflective lesions were a thickening of the region of the inner and outer segments (Fig. 2C) with the presence of an amorphous reflective zone, from the outer edge of the ONL to the RPE resulting in a loss of the reflective bands that are normally present: the external limiting membrane, ellipsoid zone, and interdigitation zone. At this stage, there was often an undulating appearance of the distal edge of the ONL. With progression, hyperreflective lesions developed in the AC and expanded, while the affected visual streak remained hyporeflective. *RDH5^−^^/^^−^* cats with hyperreflective lesions in the AC had outer retinal thinning visible on SD-OCT imaging and quantifiable by heat mapping (Fig. 2). These differences in retinal appearance and thickness are consistent across individual *RDH5^−^^/^^−^* cats at similar stages of disease. Outside of the visual streak and AC regions, retinal appearance is comparable with that of control cat retinas. The histological findings correlating with these changes in appearance and of SD-OCT cross-sectional imaging are described elsewhere in this article.

**Figure 3.**
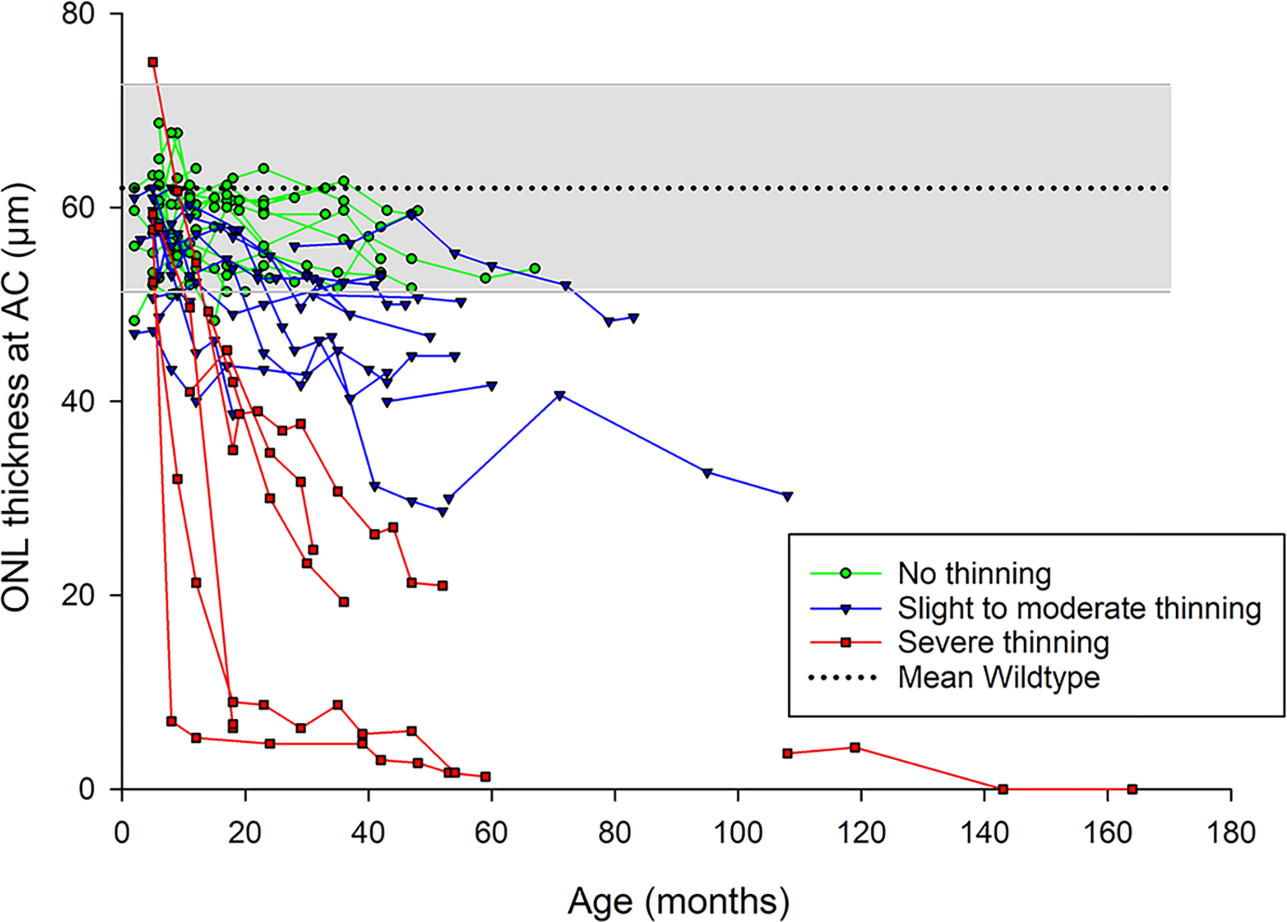
**Changes in the AC ONL thickness with age.** Each line represents an individual cat. The lines are color coded to represent severity of AC degeneration. The *dotted black line* is the mean ONL thickness of the AC in control cats and the *gray* *shaded area* represents the control mean ± 2 standard deviations.

The ONL thickness of *RDH5^−^^/^^−^* cats at the AC ranged between 0 µm and 75.0 µm, depending on the presence or absence of AC degeneration (Table 2). Figure 3 shows the progression of AC degeneration, or lack thereof, for all *RDH5^−^^/^^−^* cats as they age. *RDH5^−^^/^^−^* cats were binned into three groups based on ONL thickness at the last measurement timepoint (Table 2). Cats without ONL thinning were in group A. Cats with moderate ONL thinning were categorized as group B and those with more advanced thinning, group C. Animals that eventually progressed to group C (severe AC degeneration), typically showed hyporeflectivity of the visual streak and AC by 6 months of age and had severe thinning of the AC ONL by approximately 2 years of age. Those that advanced to group B (intermediate AC degeneration), but not into group C, typically showed fundus abnormalities by approximately 2 years of age. *RDH5^−^^/^^−^* cats with severe AC degeneration were predominantly female, with five of the seven with this level of degeneration being female. In contrast, those with no degeneration were predominantly male (9 of 11).

**Table 2.** ONL Thickness in the AC Showing Control Cats and Each Degeneration Group of *RDH5^−^^/^^−^* Cats

Group	AC Degeneration	Mean ONL Thickness of AC (µm)	Range	Standard Deviation	No.
WT	N/A	62	[56.3–71.0]	5.3	14
A	None to mild	57.2	[51.7–64.0]	5.1	17
B	Intermediate	43.0	[28.7–50.3]	7.7	11
C	Severe	10.7	[0–24.67]	10.6	7

Average ONL thickness at last age measured (µm).

### Diurnal Changes in Retinal Hyporeflective Lesions

**Figure 4.**
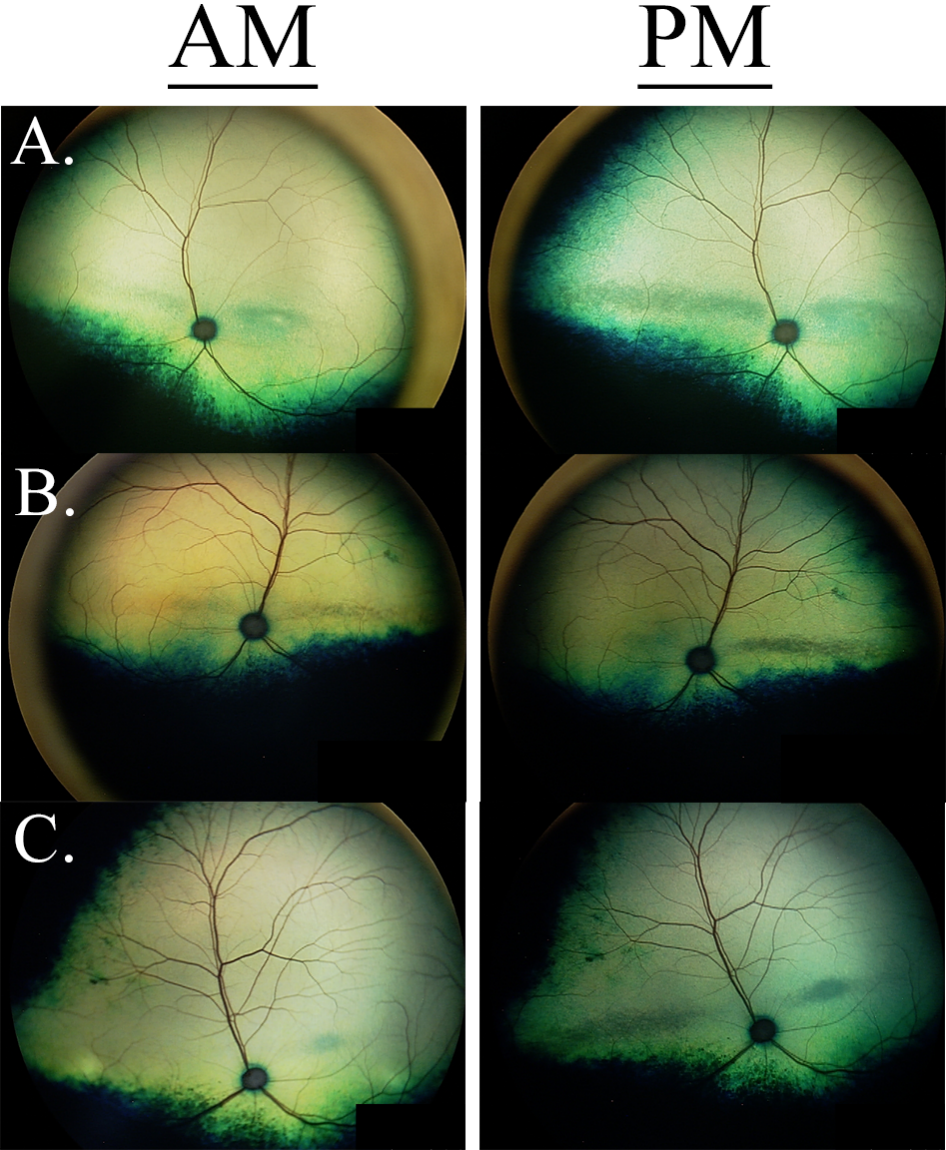
**Diurnal difference in visual streak and AC hyporeflectivity in *RDH5^−^^/^^−^* cats.** Color fundus photograph taken approximately 1 hour after light onset (AM) and midafternoon (PM) with identical camera settings following a day in normal light conditions. Photos in the same row are of the same cat. (**A**) *RDH5^−^^/^^−^* cat #19-117, (**B**) *RDH5^−^^/^^−^* cat #20-052, and (**C**) *RDH5^−^^/^^−^* cat #23-038.

To investigate diurnal changes in the appearance of the tapetal hyporeflective lesion, affected *RDH5^−^^/^^−^* cats were imaged in the morning (approximately 1 hour after lights on) and again in the afternoon after a day spent in normal room lighting (*n* = 4 *RDH5^−^^/^^−^* cats). For each *RDH5^−^^/^^−^* cat examined, the color fundus images taken in the afternoon showed a denser and more extensive horizontal hyporeflective band than when imaged in the morning. Representative fundus images are shown in Figure 4.

### Electroretinography

#### *RDH5^−/−^* Cats Have Slowed Rod Recovery Only Following Light Exposure

**Figure 5.**
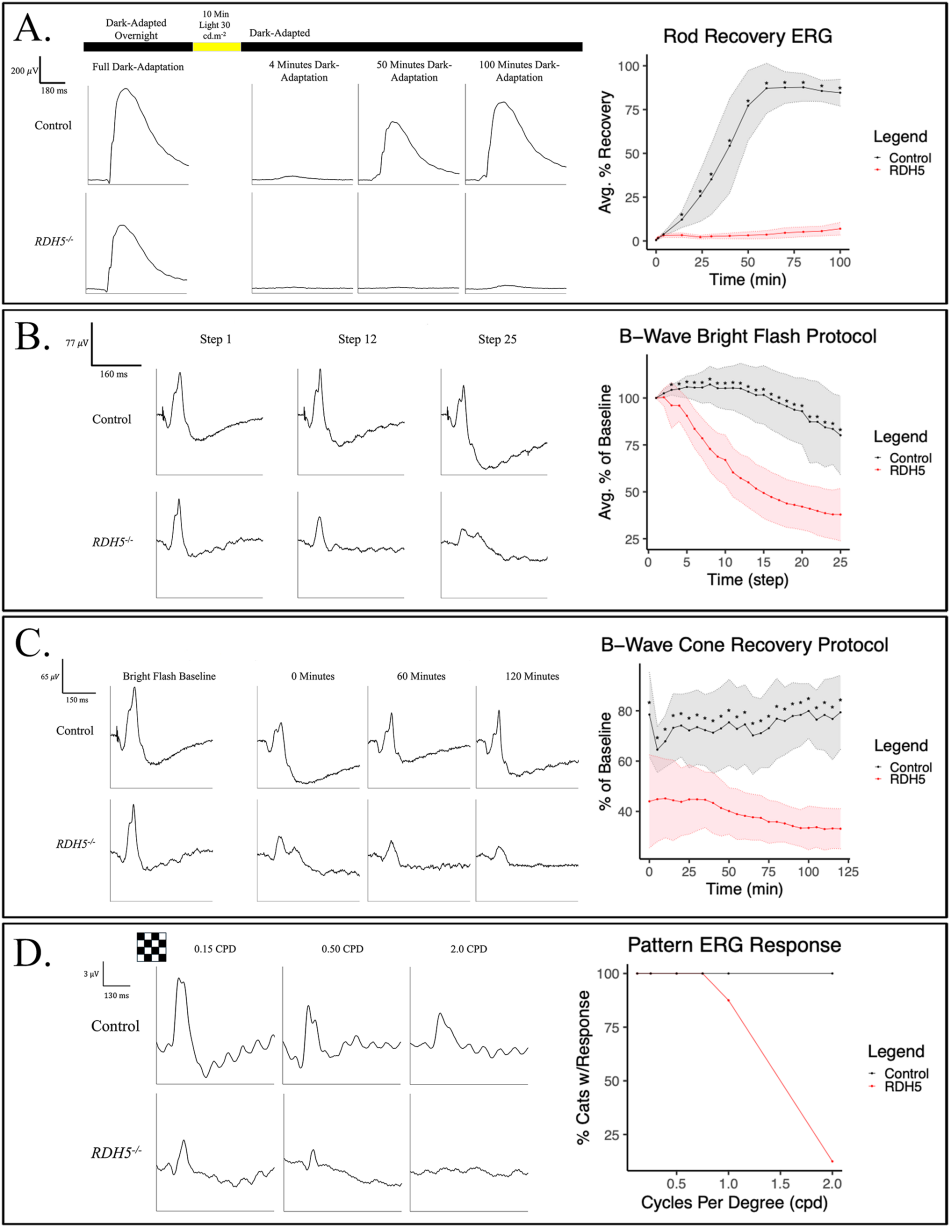
**ERG comparisons between WT and *RDH5^−^^/^^−^* cats.** The *shaded portions* in the plots on either side of the lines represent error and a * denotes significance. Waveforms are from representative individuals. (**A**) Recovery of dark adaptation ERG protocol waveform comparisons. Representative waveforms from *RDH5^−^^/^^−^* cat #18-142. (**B**) Bright flash ERG protocol waveform comparisons. Representative waveforms from *RDH5^−^^/^^−^* cat #19-117. (**C**) Cone recovery ERG protocol waveform comparisons. Representative waveforms from *RDH5^−^^/^^−^* cat #19-117. (**D**) pERG waveforms in response to a checkerboard stimulus. Representative waveforms from *RDH5^−^^/^^−^* cat #16-063.

The overnight dark-adapted ERG luminance series showed no significant differences in amplitudes between *RDH5^−^^/^^−^* cats and controls nor between the AC degeneration groups. This finding highlights how, given time, rod recovery can occur in *RDH5^−^^/^^−^* cats. However, modeling of the waveforms showed that *RDH5^−^^/^^−^* cats had a significantly higher sensitivity factor (*LogS*) and lower *t_d_* compared with the WT cats. As previously described,^21^ the recovery of the rod ERG following exposure to a rod-suppressing background light was markedly impaired in all of the *RDH5^−^^/^^−^* cats tested (rod dark-adaptation protocol). The b-wave amplitudes of WT cats in response to the International Society for Clinical Electrophysiology of Vision rod luminance (0.01 cd⋅s/m^2^) returned toward the full dark-adapted baseline amplitudes obtaining approximately 80% recovery of baseline amplitude over the 100-minute protocol. In contrast, the rod recovery of *RDH5^−^^/^^−^* cats was markedly impaired, with the b-wave amplitudes only reaching an average of approximately 7% of baseline over the 100 minutes (Fig. 5A). There was no difference between the AC degeneration groups.

#### *RDH5* ^−/−^ Cats Have Impaired Cone Function

The light-adapted single-flash ERGs of the *RDH5^−^^/^^−^* cats had significantly lower a-wave amplitudes than controls for the stronger flashes (2.5, 7.2, and 23 cd⋅s/m^2^) (*P* < 0.05). The other amplitudes and peak times were not different from controls. The *RDH5^−^^/^^−^* cats had lower amplitudes (*P* = 0.026) and shorter peak times (*P* < 0.001) for the light-adapted 33-Hz flicker compared with controls. There were no differences of any parameters when comparing *RDH5^−^^/^^−^* AC degeneration groups.

**Figure 6.**
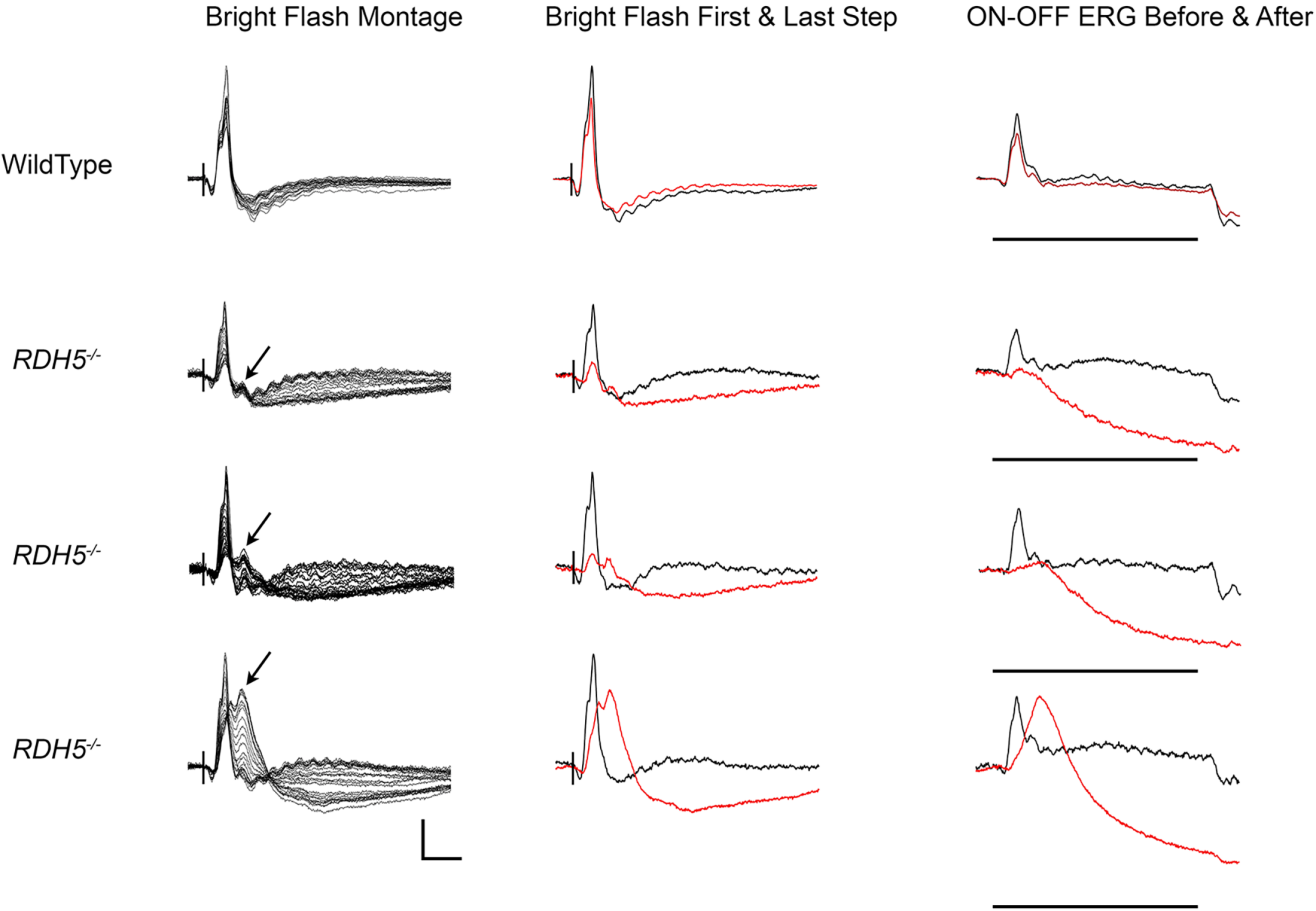
**The bright flash protocol leads to impaired cone function in *RDH5^−^^/^^−^* cats.** The *top row* is a representative WT control. The *second*, *third*, and *fourth rows* are *RDH5^−^^/^^−^* cats showing differing responses. The *first column* is a montage of all the ERGs recorded over the protocol. The second column shows the baseline ERG (*black*) and final ERG (*red*) at the end of the protocol. The final column is the ON–OFF (long flash) ERG. The *black tracing* represents a recording obtained before the bright flash protocol and the *red tracing* represents the recording at the end of the protocol. All WT controls showed a small diminution of b-wave amplitude (also see Fig. 5B), which was also reflected in the ON–OFF response, but no shape or timing alterations to the waveform. The *RDH5^−^^/^^−^* cats showed a range of changes to the photopic ERG over the course of the protocol as shown by the three examples here. Typically, there was a reduction in b-wave amplitude with variably increased peak time. Varying sizes of i-wave became apparent (*arrows*). For the ON–OFF ERG the on response varied depending on the degree of i-wave formation. With a small i-wave the ON-response was markedly reduced and with increasing i-wave it was larger and obviously delayed. There was a decreasing plateau stage and a diminished negative off response in each case. The size bars are 20 µV and 50 ms. For the short flash ERGs the timing of the flash is shown as a small vertical bar. For the ON–OFF ERGs the flash duration (250 ms) is indicated by the line beneath the ERG tracing.

To assess cone function following strong light stimulation, the “bright flash protocol” was performed. This exposed the retina to repeated strong flashes with cone ERG responses recorded between these flashes. The WT cats had a small decrease in a- and b-wave amplitudes over the duration of the protocol. In contrast, the waveform of the *RDH5^−^^/^^−^* cats was altered considerably as the number of strong flash exposures increased (Fig. 5B). The b-wave amplitude of the *RDH5^−^^/^^−^* cats was markedly reduced over the duration of the protocol and most developed a positive post b-wave waveform at about 50 ms. This timing is consistent with that of the ERG i-wave. ERG i-waves originate from retinal OFF-pathways,^35^ with timing typically around 50 ms. Similar to a and b-waves, the i-wave of many animals, including cats, is said to be homologous to the i-wave of humans.^36^ The prominence of this presumed i-wave varied between cats, with Figure 6 showing the range of changes from a cat with a small i-wave to that with a much more pronounced i-wave (indicated by arrows in the figure). Additionally, there was a difference with age in the *RDH5^−^^/^^−^* cats; age group 3 had a lower b-wave amplitude than age group 1 (*P* = 0.010) and 2 (*P* < 0.001). *RDH5^−^^/^^−^* cats also had longer a- and b-wave peak times compared with their WT counterparts.

The ON–OFF ERG was used to separate ON and OFF responses to investigate the retinal pathways responsible for the profound change in ERG waveform. The ON–OFF response at the start of the protocol was very similar between WT and *RDH5^−^^/^^−^* cats. The typical waveform for cats consists of a b-wave followed by an elevated baseline with a negative lights-off response. By the end of the bright flash protocol, WT cats had an unchanged shape of ON–OFF ERG waveform, but did have a slight decrease in the amplitudes of the b-wave and the OFF response (see Fig. 6). The protocol had a marked effect on the shape of the ON–OFF waveform of *RDH5^−^^/^^−^* cats. Figure 6 shows examples of the altered waveform in three *RDH5^−^^/^^−^* cats highlighting the range of changes. The upper *RDH5^−^^/^^−^* cat only developed a small i-wave and had a marked b-wave amplitude decrease with an increased peak time. The *RDH5^−^^/^^−^* cat in the middle row had a moderate i-wave development with a very reduced b-wave after the protocol. In contrast, the cat in the lower row developed a much larger i-wave and had less diminution of the positive ON response but with a marked increase in peak time. At the end of the protocol the ON–OFF ERG of *RDH5^−^^/^^−^* cats showed a downward slope of the plateau and a much-reduced light-OFF response.

Following the bright flash protocol, changes in cone ERGs were followed (without any further bright light flashes) to assess cone recovery indicated by recovery of the a- and b-wave amplitudes over time compared with the baseline ERG recorded before the bright flash protocol. In WT controls, the a-wave amplitude remained close to the baseline and the b-wave started at about approximately 80% of baseline and then initially decreased before recovering to approximately 80% (Fig. 5C for b-wave; a-wave data not shown). The a-wave amplitude of *RDH5^−^^/^^−^* cats had a lower percent of baseline than the WT controls (approximately 60% vs approximately 100%) and showed little recovery over the time of the protocol. There was a difference in the b-wave amplitude percent recovery with age in the *RDH5^−^^/^^−^* cats. With advancing age, the percentage of baseline recovery decreased (least squares means: 46.4.%, 41.8%, 29.4%) with a significant difference between all age group combinations (1 vs. 2: *P* = 0.002; 2 vs. 3: *P* < 0.001; 1 vs. 3: *P* < 0.001). Overall, there was no significant difference in degree of recovery to baseline between AC degeneration groups.

#### Pattern ERG

In both WT and *RDH5^−^^/^^−^* cats, as the CPD increases the response to the pattern decreases. This can be seen in an overall decrease in amplitudes for both the P1 and N2 across both groups. Although all WT cats elicited a detectable response from all CPDs tested, *RDH5^−^^/^^−^* cats had a decrease in the number of cats with a recordable response of both the P1 and N2 with increasing CPDs (Fig. 5D). Only 1 of 12 *RDH5^−^^/^^−^* cats had a distinguishable response at the 2.0 CPD. While for the detectable responses the P1 and N2 amplitudes and the N2:P1 ratio were not different, *RDH5^−^^/^^−^* cats did have a significant increase in peak time compared with the WT starting at the 0.25-CPD stimulus. For both groups, the peak time increased as the CPD increased. Age and sex were not significantly associated with differences for any of the parameters tested. When comparing degeneration groups, there appeared to be a slight correlation between AC degeneration and lower P1 and N2 amplitudes (Supplementary Fig. S2).

### Histology/Transmission Electron Microscopy

**Figure 7.**
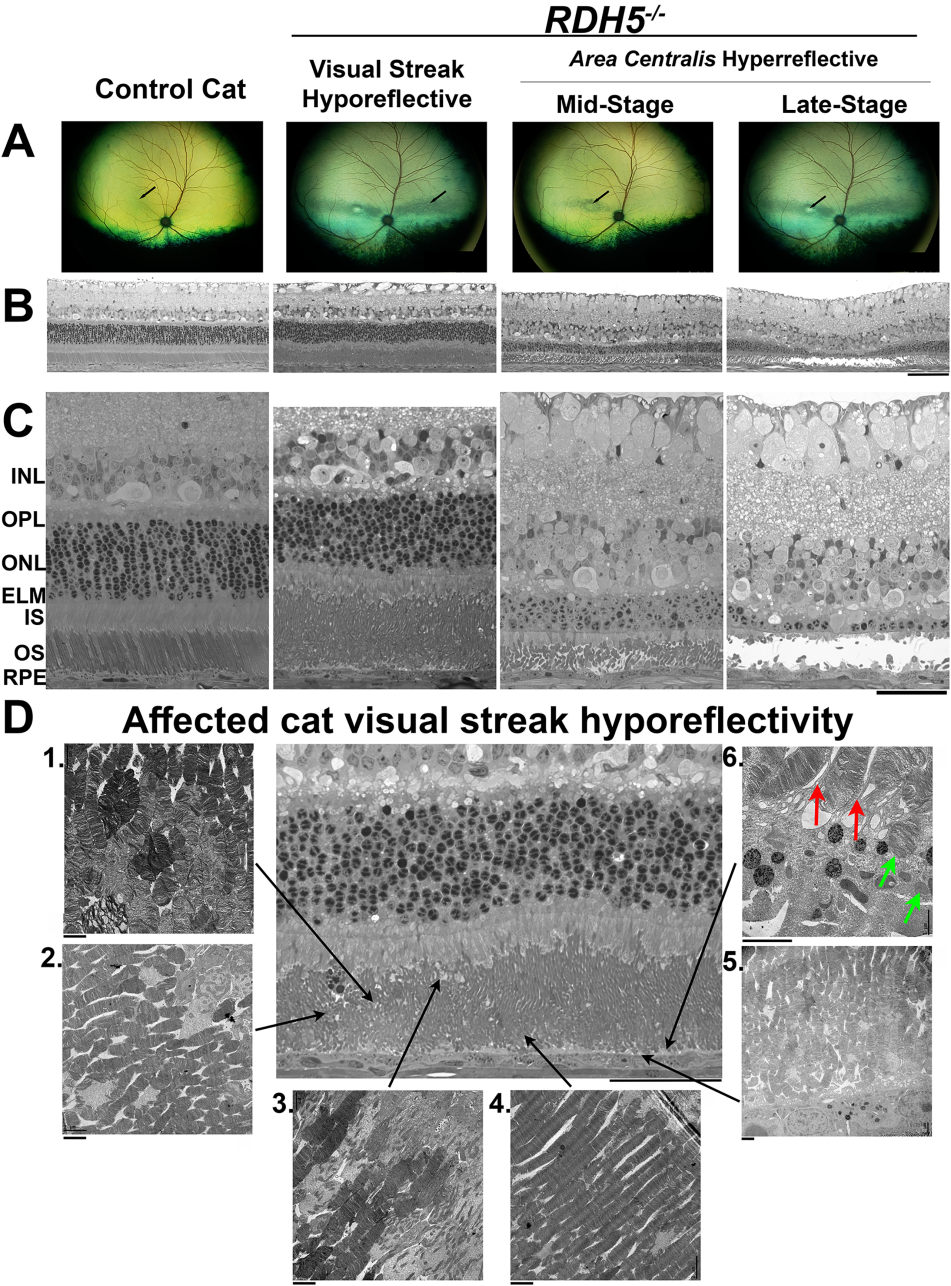
**Plastic embedded retinal semithin sections and electron micrographs at progressive disease stages.** (**A**) Fundus images with an arrow indicating where the respective sections in (**B**) and (**C**) arise from (**B**) low-magnification sections and (**C**) higher magnification of control and affected cat retinas at early-, mid-, and late-stage disease compared with a control cat retina. *Scale bar*, 100 µm. (**D**) Higher power images showing details of the early changes in the outer retina (visual streak). *Scale bar*, 100 µm. (1 to 6) Detailed electron microscopy (EM) images of the central semithin section. Overall, the OSs are elongated as shown in EM image 3 and 4. In EM image 3 there is a loss of an ordered interface between the IS and the OS. In EM image 4, the proximal portion of the OS are regularly arranged. Whereas in images 1, 2, and 5 show that there is disorganization and distortion of the distal portions of the OS. Debris has accumulated both within and between the distal ends of the distorted OS tips. EM image 6 shows that RPE processes are enveloping the distal tips of the OS (*red arrows*). Phagosome in the RPE in image 6 are indicated with *green arrows*. *Scale bar* for middle image, 50 µm; images 1–6, 2 µm. Early stage: *RDH5^−^^/^^−^* cat #0A02104314 at the visual streak. Mid stage: *RDH5^−^^/^^−^* cat #0A01681240 at the AC. Late stage: *RDH5^−^^/^^−^* cat #0A02104314 at the AC. INL, inner nuclear layer; IS, photoreceptor inner segments; ELM, external limiting membrane; OPL, outer plexiform layer; OS, photoreceptor outer segments.

Changes in retinal layer thickness detected by in vivo SD-OCT imaging were confirmed by examining plastic embedded semithin sections. Eyes with hyporeflective lesions involving the visual streak that showed thickening of the inner segment/outer segment region and amorphous increased reflectance on SD-OCT (Fig. 2B) had obvious changes on histology. Although at this stage there was no ONL layer loss, there were marked changes involving the photoreceptor inner and outer segments. The outer boundary of the ONL had a wavy appearance when compared with the control retina (Fig. 7B). This was similar to an undulating appearance of the distal boundary of the ONL in the visual streak region noted on SD-OCT imaging of eyes with visual streak hyporeflectivity (data not shown). There was also a notable irregularity in the position of the junction between the inner and outer segment regions (Figs. 7B, 7C, second column). In contrast, the WT cats’ inner segment lengths and the junctions with outer segments closely align (Figs. 7B, 7C, first column). The most striking change in *RDH5^−^^/^^−^* cats with hyporeflective lesions was outer segment elongation with distortion and disruption of the distal tips (Fig. 7D, images 1 and 2). The more proximal portions of the outer segments were relatively parallel (Fig. 7D, image 4), whereas the more distal portions were disorganized and distorted. Some of these outer segment tips were expanded with electron dense material and with similar material present between the outer segment tips (Fig. 7D, images 1, 2, and 5). RPE processes are seen enveloping the tips of the distorted photoreceptors (Fig. 7D, image 6, red arrows) with some phagosomes present (green arrows).

In the AC with midstage photoreceptor degeneration, there was ONL thinning and also distortion of and loss of length of the outer segments (Figs. 7B, 7C, third column). In advanced stages of disease and degeneration in the AC, the ONL thinned to a single layer of nuclei at the center of the lesion. Some extremely stunted inner or outer segment material was still apparent (Figs. 7B, 7C, fourth column).

### Immunohistochemistry

**Figure 8.**
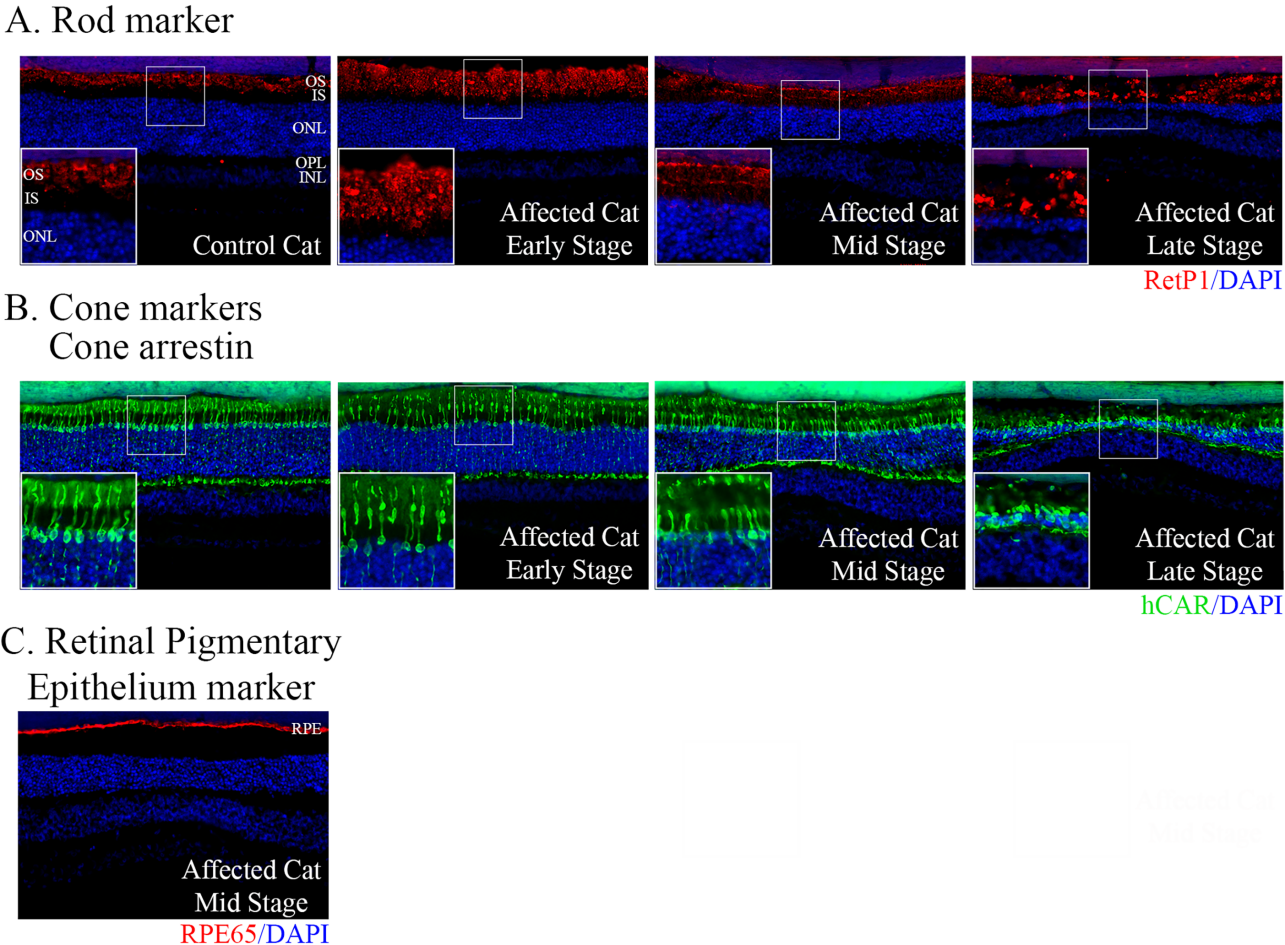
**Immunolabeling of retinal sections across the affected retinal regions at progressive disease stages.** (**A**) Rod marker (RetP1) labeling. In the early disease stage, the rod OSs were longer than those of control animals (detail shown in the *inset image*). At the mid stage disorganization and loss of OS/IS was apparent at the center of the AC and there was some mislocalization of rhodopsin to the inner segments and outer portion of the ONL. By the late stage, there was pronounced loss of rods with marked thinning of the ONL and disruption of the remaining RetP1 immunolabeled inner and OSs. (**B**) Cone arrestin (hCAR) labeling (labels entire cone cell) shows that in mid-stage disease there is reduced cone OSs hCAR labeling in the very center of the AC, but even when the ONL is thinned to one or two rows of photoreceptor nuclei, cones are still present. At the late stage of the disease, the remaining cone inner segments are stunted and distorted with little discernible OS material (**C**) RPE65 retinal pigmentary epithelium cells labeling. This demonstrates that the RPE is still present in the center of the lesion, showing that RPE loss does not precede photoreceptor loss. OS, photoreceptor outer segments; IS, photoreceptor inner segments; OPL, outer plexiform layer; INL, inner nuclear layer. Early stage: *RDH5^−^^/^^−^* cat #0A01664141. Mid stage: *RDH5^−^^/^^−^* cat #0A01681240. Late stage: *RDH5^−^^/^^−^* cat #0A02104314.

RetP1, an antibody targeting rhodopsin, labels rod outer segments in the WT cat (Fig. 8A). Early-stage lesions in affected cats showed lengthening of RetP1-labeled rod outer segments (corresponding with fundus hyporeflective lesions in the AC and visual streak). For more advanced lesions with thinning of the ONL, outer segments became more disrupted, and RetP1 mislocalization to the inner segments could be observed. (Fig. 8A, third column). With further photoreceptor degeneration, there was a loss of RetP1-labelled rod outer segments at the center of the lesion. However, even in late-stage degeneration there remained some clumps of RetP1 immunoreactive material (Fig. 8A, fourth column).

Human cone arrestin antibody labels the entire cone cell body in the cat and showed normal-appearing cones in early-stage disease that were comparable with those of the WT control (Fig. 8B, second and first columns, respectively). As photoreceptor degeneration progressed, cone outer segments became disorganized (Fig. 8B, third column). By late-stage degeneration (Fig. 8, fourth column), there was also disruption of the cone inner segments, but cone arrestin–positive cell bodies remained, even in the advanced degeneration in the center of the AC. Figure 8C demonstrates immunolabeling of the RPE with an RPE65 antibody. This staining showed that there was not a loss of RPE before photoreceptor degeneration.

## Discussion

Biallelic *RDH5* mutations in humans result in a congenital night blindness due to delayed recovery of rods from light exposure. Affected patients develop white–yellowish retinal lesions radiating out from the macula giving the condition its name, FA. The rod functional defects result from severe delays in 11-*cis*-retinal regeneration. With modern retinal imaging techniques, cone abnormalities are detected in many patients and cone involvement is now more commonly recognized as a disease characteristic.^15^ Although all FA patients experience night blindness, a subset also develops MD. One recent study showed a progressive loss of macular volume in all FA patients after the fourth decade of life.^37^

Our previously published article describing the phenotype of *RDH5* mutant cats demonstrated a lack of RDH5 function in the cats homozygous for the mutation and compared the accompanying phenotype with that of human subjects with biallelic mutations in *RDH5*. Delayed rod dark adaptation and AC degeneration (cats) or MD (humans) in a subset of affected cats and humans was observed. These phenotypic similarities between the two species highlight the *RDH5^−^^/^^−^* cat as a valuable model, more closely mimicking the phenotypic changes in affected humans than mouse models do. *Rdh5* knockout mice have milder functional changes and do not develop changes equivalent to MD, as they lack a macula-equivalent region. The current study further investigated the structural changes associated with the development of AC degeneration and further characterized the rod and cone dysfunction in the *RDH5^−^^/^^−^* cat.

Ophthalmoscopic fundus lesions in cats that go on to develop AC degeneration initially present with a horizontal hyporeflective gray-colored band in the ventral tapetal fundus corresponding with the AC and visual streak. These are retinal regions with an increased packing density of photoreceptors including a higher concentration of cones and, similar to the macula in humans, are for higher acuity vision. With progression, the lesion in the AC becomes hyperreflective, an indicator of retinal thinning. There was considerable variability between *RDH5^−^^/^^−^* cats, ranging from some not developing those changes when followed to middle age to those who developed them at a few months of age and rapidly progressed to AC retinal thinning. We hypothesize that these differences in AC degeneration are likely the cause of a genetic modifier and, thus, genomic analyses are currently underway in an attempt to identify potential genetic modifiers.

Within the colony, approximately 70% of the *RDH5^−^^/^^−^* cats that developed severe retinal thinning were female. This finding aligns with other models of retinal degeneration, where females are more severely affected. For example, in a retinitis pigmentosa mouse model, females’ ONL degenerated at a faster rate than their male counterparts. This was hypothesized to be due to the action of female-specific sex hormones.^38^ Additionally, humans studies have shown age-related MD to be more prevalent in women compared with men.^39^ Interestingly, in another study, women using postmenopausal hormones were found to be associated with a 34% higher risk of early age-related MD but 48% lower risk for the late-stage neovascular (wet) age-related MD, indicating estrogen is impacting severity of age-related MD.^40^

In vivo imaging and histology identified the retinal changes leading to these ophthalmoscopic lesions. Cross-sectional SD-OCT imaging of the retina indicated the hyporeflective fundus lesions affecting the AC and visual streak had a thickening of the region from the outer limiting membrane to the RPE with an amorphous appearance and loss of visualization of the external limiting membrane, ellipsoid zone, and interdigitation zone reflective bands. Unlike the typical straight and organized appearance of the photoreceptor outer segments, transmission electron microscopy images revealed there was a marked elongation of photoreceptor outer segments with disorganization of their distal ends in *RDH5^−^^/^^−^* cats with hyporeflectivity. This hyporeflectivity also correlates with an increase in fundus autofluorescence, likely a result of the lengthened and distorted photoreceptor outer segments.^41^ With loss of photoreceptors and tapetal fundus hyperreflectivity, there was a decrease in fundus autofluorescence, correlating with photoreceptor degeneration and hyperreflectivity of the AC. One explanation for this disorganization of the photoreceptors is a disruption to the outer segment shedding/phagocytosis process.

A proportion of the outer segment discs are phagocytosed and recycled by the RPE daily.^42^ It is likely that this process is slowed, allowing the outer segments to elongate, pushing up against the RPE and resulting in the distorted appearance of the outer segment tips (Figs. 7, 8). The hyporeflective lesions of the AC and visual streak were found to worsen over the course of the day, further supporting the suggestion that RPE phagocytosis is slowed. In cats, among other species, RPE phagocytosis has been shown to peak 1 to 2 hours after the onset of light.^43^^,^^44^ With suboptimal phagocytosis, there is likely accumulation of unphagocytosed outer segment material over the course of the day, resulting in the more pronounced hyporeflectivity apparent in the afternoon imaging session. SD-OCT, histology, and immunohistochemistry confirmed that, as disease progressed from hyporeflectivity to hyperreflectivity in the AC, both rod and cone photoreceptors were lost. A single case report described photoreceptor elongation in an FA patient on SD-OCT.^45^ The lack of other reports of FA patients with photoreceptor elongation as a precursor to MD may indicate species differences between human and cat or that impaired outer segment phagocytosis is not a major part of the pathophysiology of photoreceptor loss in FA patients. Further studies to fully elucidate the mechanism of photoreceptor loss in FA patients and *RDH5^−^^/^^−^* cats are required.

The marked delay in recovery of rod function after exposure to a bright light highlights the importance of *RDH5* in supplying 11-*cis*-retinal for normal recovery of the rod visual pigment, rhodopsin. However, once fully dark adapted, rod ERG tracings were similar to controls. The similar scotopic amplitudes, with extended dark adaptation, seen in these *RDH5^−^^/^^−^* cats, matches the reported human FA phenotype.^15^ When analyzing the rod-driven portion of the a-wave using the Birch and Hood version of the Lamb and Pugh rod phototransduction model, the *RDH5^−^^/^^−^* cats had a higher sensitivity value (*LogS*) and a lower time delay (*t_d_*) compared with WT cats. *LogS* is an indicator of rod photoreceptor sensitivity. Typically, *LogS* values would be higher in WT animals compared with those with retinal insufficiencies.^46^ Therefore, the higher sensitivity value in the *RDH5^−^^/^^−^* cats compared with WT is interesting and further study is required to elucidate the cause of these differences Similarly, the time delay parameter indicates the time from flash onset to the beginning of the a-wave,^47^ so a lower time delay in the *RDH5^−^^/^^−^* cats compared with WT was unexpected and further study is needed to determine the cause of this abnormality.

In humans, photopic flicker responses are often the first indicators of cone dysfunction.^48^ The *RDH5^−^^/^^−^* cats resemble this by having significantly lower cone flicker amplitudes, but their significantly faster responses compared with WT were unexpected and do not match that of humans. Further investigation is needed to determine the cause of shorter peak times in *RDH5^−^^/^^−^* cats. The significant decrease in cone ERG responses between *RDH5^−^^/^^−^* and WT cats indicates that *RDH5^−^^/^^−^* cats are much more susceptible to strong repeated cone stimulation. Due to the lack of functional RDH5, there is already a lower level of 11-*cis*-retinal available to cones. Although the cone-exclusive intraretinal visual cycle does supply 11-*cis*-retinol that cones can convert to 11-*cis*-retinal, it appears that this supply may become depleted, resulting in abnormal cone phototransduction and thus altered ERG waveforms in the *RDH5^−^^/^^−^* cats.

After cessation of the repeated strong flash stimulation, the a- and b-wave amplitudes as a percentage of the baseline response of *RDH5^−^^/^^−^* cats continued to decrease in the “cone recovery protocol.” In contrast, the WT cats’ responses remained relatively constant, reflecting the ability of WT cones to begin to replenish 11-*cis*-retinal supply once exposure to the strong flash was removed. The bright flash a-wave amplitude was significantly different between all degeneration groups at a large portion of the steps. Interestingly, the most severely degenerated group has higher responses compared with the other groups. As the *RDH5^−^^/^^−^* cat's b-wave amplitude decreased and peak time became greater during the cone bright flash protocol, an i-wave appeared in most of the animals. The degree of b-wave reduction and delay and of i-wave amplitude varied between individual cats, but this did not correlate with AC degeneration status. As the cone recovery protocol was continued and the bright light stimulus removed, the i-wave, thought to arise from inner retinal responses, decreased in amplitude. It is not clear which changes in cone function result in amplitude changes of the i-wave, and the appearance of the i-wave warrants further investigation.

The ON–OFF ERG can be used to separate the “light-on” from the “light-off” response that in a short flash ERG are superimposed. The marked decrease in ON-response seen in the ON–OFF ERG of *RDH5^−^^/^^−^* cats may indicate that the ON-pathways were more impacted than the OFF-pathway.

In addition to the *RDH5^−^^/^^−^* cones’ increased susceptibility to 11-*cis*-retinal depletion, the AC cones exhibit a loss of sensitivity as shown by pERG results. With pERGs, despite not having a significant difference in P1 amplitudes, *RDH5^−^^/^^−^* cats failed to generate a detectable response to higher CPDs. Starting at the 0.50 CPD, *RDH5^−^^/^^−^* cats showed a marked increase in peak time. As the P1 amplitude is an indicator of cone system function within the AC region, the loss of sensitivity and the delayed peak indicates an overall AC cone dysfunction compared with WT. It is possible that the differences in the P1 amplitude did not reach significance due to insufficient statistical power. Another possibility is that, at the lower CPDs, *RDH5^−^^/^^−^* cats are able to elicit a response similar to WTs. This ability, however, is extinguished at the higher CPDs. The N2 amplitude is generated from retinal ganglion cell activity and a decrease in amplitude suggests altered retinal ganglion cell function. While compiled *RDH5^−^^/^^−^* responses result in comparable responses between normal and affected animals for both the N2 amplitude and the N2:P1 ratio, there is a marked reduction between the two groups, although this does not reach statistical significance (Supplementary Fig. S2). These findings may indicate variable retinal ganglion cell dysfunction in *RDH5^−^^/^^−^* cats. This idea is supported by the variable presence of the i-wave in photopic ERGs under strong light stimulation, a response thought to reflect a late contribution from retinal ganglion cells, as described elsewhere in this article. One possible explanation for this inner retinal dysfunction described in the *RDH5^−^^/^^−^* cats is that the photoreceptor dysfunction in these animals modifies the transfer of signal via ON and OFF pathways. This then results in an alteration in waveforms originating from inner retinal neurons.

Although the *RDH5^−^^/^^−^* cat has previously been characterized as having rod dysfunction and abnormalities in the AC region, cone function and the effect of AC degeneration overall had not been studied. It is clear that, despite having a *RDH5* mutation that primarily impacts rods, this model recapitulates the cone dysfunction that can be seen in human patients as well. This impact on cones appears to be variable and reflects aspects of reported FA disease. For the majority of ERG measures, AC degeneration status did not have a significant impact on ERG responses. The generalized rod and cone dysfunction was present regardless of whether there was AC degeneration or not. As the AC represents only a small proportion of the retina, degeneration in this small region is not expected to alter full-field ERG recordings if the functional changes were not pan-retinal. Overall, this large-animal cat model is important for future testing of gene therapies and could provide crucial information on the potential impacts of modifier genes, highlighting potential candidates for humans with FA.

## Supplementary Material

Supplement 1

## References

[1] Filipek S, Stenkamp RE, Teller DC, Palczewski K. G protein-coupled receptor rhodopsin: a prospectus. *Annu Rev Physiol*. 2003; 65(1): 851–879. 12471166 10.1146/annurev.physiol.65.092101.142611 PMC1435697

[2] Daruwalla A, Choi EH, Palczewski K, Kiser PD. Structural biology of 11-cis-retinaldehyde production in the classical visual cycle. *Biochem J*. 2018; 475(20): 3171–3188. 30352831 10.1042/BCJ20180193 PMC6440480

[3] Kiser PD, Palczewski K. Retinoids and retinal diseases. *Annu Rev Vis Sci*. 2016; 2: 197–234. 27917399 10.1146/annurev-vision-111815-114407 PMC5132409

[4] Sato S, Kefalov VJ. The retina-based visual cycle. *Annu Rev Vis Sci*. 2024; 10(1): 293–321. 38724080 10.1146/annurev-vision-100820-083937

[5] Kaylor JJ, Frederiksen R, Bedrosian C, et al. Identification of RDH12 as the protein that allows cones to access the retinal visual cycle. *Invest Ophthalmol Vis Sci*. 2024; 65(7): 1328.

[6] Parker RO, Crouch RK. Retinol dehydrogenases (RDHs) in the visual cycle. *Exp Eye Res*. 2010; 91(6): 788–792. 20801113 10.1016/j.exer.2010.08.013 PMC3065351

[7] Yamamoto H, Simon A, Eriksson U, Harris E, Berson EL, Dryja TP. Mutations in the gene encoding 11-cis retinol dehydrogenase cause delayed dark adaptation and fundus albipunctatus. *Nat Genet*. 1999; 22(2): 188–191. 10369264 10.1038/9707

[8] Gonzalez-Fernandez F, Kurz D, Newman S, Conway BP, Han DP, Khani SC. 11-cis retinol dehydrogenase mutations as a major cause of the congenital night-blindness disorder known as fundus albipunctatus. *Mol Vis*. 1999; 5: 41. 10617778

[9] Sergouniotis PI, Sohn EH, Li Z, et al. Phenotypic variability in RDH5 retinopathy (fundus albipunctatus). *Ophthalmology*. 2011; 118(8): 1661–1670. 21529959 10.1016/j.ophtha.2010.12.031

[10] Farjo KM, Moiseyev G, Takahashi Y, Crouch RK, Ma J-x. The 11-cis-retinol dehydrogenase activity of RDH10 and its interaction with visual cycle proteins. *Invest Ophthalmol Vis Sci*. 2009; 50(11): 5089–5097. 19458327 10.1167/iovs.09-3797

[11] Kim TS, Maeda A, Maeda T, et al. Delayed dark adaptation in 11-cis-retinol dehydrogenase-deficient mice: a role of RDH11 in visual processes in vivo. *J Biol Chem*. 2005; 280(10): 8694–8704. 15634683 10.1074/jbc.M413172200 PMC1351245

[12] Katagiri S, Hayashi T, Nakamura M, et al. RDH5-related fundus albipunctatus in a large Japanese cohort. *Invest Ophthalmol Vis Sci*. 2020; 61(3): 1–11. 10.1167/iovs.61.3.53 PMC7401827 32232344

[13] Nakamura M, Miyake Y. Macular dystrophy in a 9-year-old boy with fundus albipunctatus. *Am J Ophthalmol*. 2002; 133(2): 278–280. 11812441 10.1016/s0002-9394(01)01304-6

[14] Sekiya K, Nakazawa M, Ohguro H, et al. Long-term fundus changes due to fundus albipunctatus associated with mutations in the RDH5 Gene. *Arch Ophthalmol*. 2003; 121(7): 1057–1059. 10.1001/archopht.121.7.1057-b 12860821

[15] Skorczyk-Werner A, Pawłowski P, Michalczuk M, et al. Fundus albipunctatus: review of the literature and report of a novel RDH5 gene mutation affecting the invariant tyrosine (p.Tyr175Phe). *J Appl Genet*. 2015; 56(3): 317–327. 25820994 10.1007/s13353-015-0281-x PMC4543405

[16] Pras E, Pras E, Reznik-Wolf H, et al. Fundus albipunctatus: novel mutations and phenotypic description of Israeli patients. *Mol Vis*. 2012; 18: 1712–1718. 22815624 PMC3399783

[17] AlShawabkeh Ma, Alryalat SA, Abu-Ameerh M, Sallam AA. Clinical review of retina and vitreous diseases: part II. In: Alryalat SA, ed. *Ophthalmology Board and FRCS Part 2 Exams: Eye Yield Clinical*. London: Springer Nature; 2025: 431–455.

[18] Berman K, Brodaty H. Psychosocial effects of age-related macular degeneration. *Int Psychogeriatr*. 2006; 18(3): 415–428. 16466594 10.1017/S1041610205002905

[19] Nakamura M, Skalet J, Miyake Y. RDH5 gene mutations and electroretinogram in fundus albipunctatus with or without macular dystrophy. *Doc Ophthalmol*. 2003; 107(1): 3–11. 12906118 10.1023/a:1024498826904

[20] Driessen C, Winkens HJ, Hoffmann K, et al. Disruption of the 11-cis-retinol dehydrogenase gene leads to accumulation of cis-retinols and cis-retinyl esters. *Mol Cell Biol*. 2000; 20(12): 4275–4287. 10825191 10.1128/mcb.20.12.4275-4287.2000 PMC85795

[21] Occelli LM, Daruwalla A, De Silva SR, et al. A large animal model of RDH5-associated retinopathy recapitulates important features of the human phenotype. *Hum Mol Genet*. 2021; 31(8): 1263–1277. 10.1093/hmg/ddab316 PMC9029234 34726233

[22] Mowat FM, Gervais KJ, Occelli LM, et al. Early-onset progressive degeneration of the area centralis in RPE65-deficient dogs. *Invest Ophthalmol Vis Sci*. 2017; 58(7): 3268–3277. 28662231 10.1167/iovs.17-21930

[23] Occelli LM, Tran NM, Narfström K, Chen S, Petersen-Jones SM. Crx^Rdy^ cat: a large animal model for CRX-associated Leber congenital amaurosis. *Invest Ophthalmol Vis Sci*. 2016; 57(8): 3780–3792. 27427859 10.1167/iovs.16-19444 PMC4960999

[24] Naka K, Rushton W. S-potentials from luminosity units in the retina of fish (Cyprinidae). *J Physiol*. 1966; 185(3): 587–599. 5918060 10.1113/jphysiol.1966.sp008003 PMC1395832

[25] Naka K, Rushton W. The generation and spread of S-potentials in fish (Cyprinidae). *J Physiol*. 1967; 192(2): 437. 6050157 10.1113/jphysiol.1967.sp008308 PMC1365565

[26] Evans LS, Peachey NS, Marchese AL. Comparison of three methods of estimating the parameters of the Naka-Rushton equation. *Doc Ophthalmol*. 1993; 84(1): 19–30. 8223107 10.1007/BF01203279

[27] *R: A Language and Environment for Statistical Computing* . Vienna: R Foundation for Statistical Computing; 2022. http://www.R-project.org/.

[28] Brigell M, Jeffrey BG, Mahroo OA, Tzekov R. ISCEV extended protocol for derivation and analysis of the strong flash rod-isolated ERG a-wave. *Doc Ophthalmol*. 2020; 140(1): 5–12. 31902035 10.1007/s10633-019-09740-4

[29] Hood DC, Birch DG. Assessing abnormal rod photoreceptor activity with the a-wave of the electroretinogram: applications and methods. *Doc Ophthalmol*. 1996; 92(4): 253–267. 9476593 10.1007/BF02584080

[30] Hood DC, Birch DG. The A-wave of the human electroretinogram and rod receptor function. *Invest Ophthalmol Vis Sci*. 1990; 31(10): 2070–2081. 2211004

[31] Raybaut P . Spyder-documentation. 2009; 769. Available at: pythonhosted.org.

[32] Mowat FM, Breuwer AR, Bartoe JT, et al. RPE65 gene therapy slows cone loss in Rpe65-deficient dogs. *Gene Ther*. 2013; 20(5): 545–555. 22951453 10.1038/gt.2012.63

[33] Bates D, Mächler M, Bolker B, Walker S. Fitting linear mixed-effects models using lme4. *J Stat Softw*. 2015; 67: 1–48.

[34] Kuznetsova A, Brockhoff PB, Christensen RHB. lmerTest package: tests in linear mixed effects models. *J Stat Softw*. 2017; 82(13): 1–26.

[35] Xu Z, Tan JK, Vetrivel K, et al. The electroretinogram I-wave, a component originating in the retinal OFF-pathway, associates with a myopia genetic risk polymorphism. *Invest Ophthalmol Vis Sci*. 2024; 65(13): 21. 10.1167/iovs.65.13.21 PMC11562975 39530998

[36] Rosolen SG, Rigaudière F, LeGargasson JF, et al. Comparing the photopic ERG i-wave in different species. *Vet Ophthalmol*. 2004; 7(3): 189–192. 15091327 10.1111/j.1463-5224.2004.04022.x

[37] Bianco L, Antropoli A, Benadji A, et al. RDH5 and RLBP1-associated inherited retinal diseases: refining the spectrum of stationary and progressive phenotypes. *Am J Ophthalmol*. 2024; 267: 160–171. 38945349 10.1016/j.ajo.2024.06.016

[38] Rowe AA, Velasquez MJ, Aumeier JW, et al. Female sex hormones exacerbate retinal neurodegeneration. *Sci Adv*. 2025; 11(15): eadr6211. 40215317 10.1126/sciadv.adr6211 PMC11988432

[39] Javitt JC, Zhou Z, Maguire MG, Fine SL, Willke RJ. Incidence of exudative age-related macular degeneration among elderly Americans. *Ophthalmology*. 2003; 110(8): 1534–1539. 12917168 10.1016/S0161-6420(03)00495-0

[40] Feskanich D, Cho E, Schaumberg DA, Colditz GA, Hankinson SE. Menopausal and reproductive factors and risk of age-related macular degeneration. *Arch Ophthalmology*. 2008; 126(4): 519–524. 10.1001/archopht.126.4.519 18413522

[41] Matsumoto H, Kishi S, Sato T, Mukai R. Fundus autofluorescence of elongated photoreceptor outer segments in central serous chorioretinopathy. *Am J Ophthalmol*. 2011; 151(4): 617–623. e611. 21257153 10.1016/j.ajo.2010.09.031

[42] Strauss O, Helbig H. The function of the retinal pigment epithelium. In: Levin LA, Nilsson SFE, Hoeve JV, Wu SM, eds. *Adler's Physiology of the Eye*. 11th ed. New York: Elsevier; 2011: 325–332:chap 13.

[43] DeVera C, Tosini G. Circadian analysis of the mouse retinal pigment epithelium transcriptome. *Exp Eye Res*. 2020; 193: 107988. 32105725 10.1016/j.exer.2020.107988 PMC7372111

[44] Fisher SK, Pfeffer BA, Anderson DH. Both rod and cone disc shedding are related to light onset in the cat. *Invest Ophthalmol Vis Sci*. 1983; 24(7): 844–856. 6683265

[45] Sobol EK, Deobhakta A, Wilkins CS, et al. Fundus albipunctatus photoreceptor microstructure revealed using adaptive optics scanning light ophthalmoscopy. *Am J Ophthalmol Case Rep*. 2021; 22: 101090. 33981912 10.1016/j.ajoc.2021.101090 PMC8082516

[46] Pasmanter N, Occelli LM, Komáromy AM, Petersen-Jones SM. Use of extended protocols with nonstandard stimuli to characterize rod and cone contributions to the canine electroretinogram. *Doc Ophthalmol*. 2022; 144(2): 81–97. 35247111 10.1007/s10633-022-09866-y PMC10426558

[47] Pasmanter N, Petersen-Jones SM. Characterization of scotopic and mesopic rod signaling pathways in dogs using the ON–OFF electroretinogram. *BMC Vet Res*. 2022; 18(1): 422. 36463174 10.1186/s12917-022-03505-z PMC9719241

[48] Miyake Y, Shiroyama N, Sugita S, Horiguchi M, Yagasaki K. Fundus albipunctatus associated with cone dystrophy. *Br J Ophthalmol*. 1992; 76(6): 375–379. 1622952 10.1136/bjo.76.6.375 PMC504291

