# Supplementary material for: Functional and Structural Characterization of a Large Animal Model of RDH5-Associated Retinopathy
Source: Transl Vis Sci Technol. 2026 Aug 5;15(8):2. doi: 10.1167/tvst.15.8.2 (PMC13450645; doi:10.1167/tvst.15.8.2)
Supplement: Supplementary file 1 [file tvst-15-8-2_s001.docx]

***Table S1. Age and Sex of Study Animals for each ERG protocol.*** The number of *RDH5^-/-^* cats and wildtype (WT) timepoints and the age and sex of those animals at the respective timepoints. They are divided by stage of *area centralis* degeneration based on outer nuclear layer (ONL) thickness by SD-OCT compared to WT cats: early (no thinning); mid (thinning up to ~50% loss) and late (more than 50% ONL loss). The age groups were 1: <1.5 years; 2: 1.5-3 yrs and 3: >3 yrs.

|  | ***Rod Dark Adaptation*** | | | | | | | |
| --- | --- | --- | --- | --- | --- | --- | --- | --- |
|  | **Early (A)** | | **Mid (B)** | | **Late (C)** | | **WT** | |
| ***Age Group*** | **M** | **F** | **M** | **F** | **M** | **F** | **M** | **F** |
| **1** | 19 | 10 | 1 | 3 | 0 | 1 | 0 | 4 |
| **2** | 1 | 0 | 3 | 2 | 0 | 3 | 0 | 1 |
| **3** | 2 | 1 | 1 | 2 | 1 | 1 | 1 | 0 |
|  | ***Bright Flash*** | | | | | | | |
| **1** | 18 | 11 | 0 | 3 | 0 | 1 | 0 | 4 |
| **2** | 2 | 0 | 4 | 3 | 0 | 2 | 0 | 1 |
| **3** | 2 | 0 | 1 | 3 | 0 | 2 | 1 | 2 |
|  | ***Cone Recovery*** | | | | | | | |
| **1** | 17 | 11 | 0 | 3 | 0 | 1 | 0 | 4 |
| **2** | 1 | 0 | 4 | 3 | 0 | 2 | 0 | 1 |
| **3** | 2 | 0 | 1 | 3 | 0 | 2 | 1 | 2 |
|  | ***Scotopic (dark-adapted)*** | | | | | | | |
| **1** | 19 | 12 | 1 | 3 | 0 | 2 | 1 | 3 |
| **2** | 1 | 4 | 6 | 8 | 0 | 2 | 1 | 3 |
| **3** | 2 | 1 | 2 | 4 | 1 | 1 | 0 | 1 |
|  | ***Photopic (light adapted)*** | | | | | | | |
| **1** | 19 | 12 | 1 | 3 | 0 | 2 | 0 | 3 |
| **2** | 1 | 4 | 6 | 8 | 0 | 2 | 1 | 4 |
| **3** | 2 | 1 | 2 | 4 | 1 | 1 | 1 | 0 |
|  | ***Pattern*** | | | | | | | |
|  | ***RDH5^-/-^* M** | | | ***RDH5^-/-^* F** | | | **WT M** | **WT F** |
| **1** | 0 | | | 0 | | | 0 | 3 |
| **2** | 1 | | | 1 | | | 0 | 1 |
| **3** | 5 | | | 5 | | | 1 | 2 |

***Table S2. Study Animal Identifying Information and Use.*** *RDH5^-/-^* cats and their corresponding sex, degeneration status, and procedures performed.

| **Identifying #** | **Sex** | ***AC* Degeneration*** | **ERG** | **OCT** | **IHC** | **EM** |
| --- | --- | --- | --- | --- | --- | --- |
| 16-063 | F | C | X | X |  |  |
| 20-039 | F | B | X | X |  |  |
| 20-040 | F | B | X | X |  |  |
| 21-061 | F | B | X | X |  |  |
| 21-036 | F | A | X | X |  |  |
| 20-054 | F | C | X | X |  |  |
| 20-055 | M | B | X | X |  |  |
| 20-012 | M | A | X | X |  |  |
| 18-142 | F | B | X | X |  |  |
| 21-038 | M | A | X | X |  |  |
| 0A02104314 | F | C |  | X | X | X |
| 19-118 | M | B | X | X |  |  |
| 19-117 | F | B | X | X |  |  |
| 20-052 | F | C | X | X |  |  |
| 21-065 | M | A | X | X |  |  |
| 0A01681240 | F | C |  | X | X | X |
| 20-056 | M | A | X | X |  |  |
| 23-039 | F | A |  | X |  |  |
| 17-030 | M | A |  | X |  |  |
| 17-029 | M | A |  | X |  |  |
| 21-037 | M | A | X | X |  |  |
| 21-064 | M | A | X | X |  |  |
| 23-038 | F | B |  | X |  |  |
| 18-046 | M | B |  | X |  |  |
| 20-053 | F | B | X | X |  |  |
| 21-062 | F | A | X | X |  |  |
| 0A01664141 | M | C |  | X | X |  |
| 20-057 | M | B | X | X |  |  |
| 10-105 | M | C | X | X |  |  |
| 23-040 | F | A |  | X |  |  |

*A = no *AC* thinning; B= moderate *AC* thinning; C= marked *AC* thinning

***Figure S1. Measuring the Pattern ERG Waveforms.*** P1 is measured from the trough of the initial negative waveform to the peak of P1 (blue line). N2 is measured from the peak of P1 to the trough of N2 (purple line).

***Figure S2. Pattern ERG P1 and N2.*** Comparison between wildtype, *RDH5^-/-^* cats with no to mild, intermediate, and severe *area centralis* degeneration for the pattern ERG **(A)** P1 amplitudes and **(B)** N2 amplitudes.
